# LncRNA HCP5 promotes follicular thyroid carcinoma progression via miRNAs sponge

**DOI:** 10.1038/s41419-018-0382-7

**Published:** 2018-03-07

**Authors:** Leilei Liang, Jingchao Xu, Meng Wang, Gaoran Xu, Ning Zhang, Guangzhi Wang, Yongfu Zhao

**Affiliations:** Department of General Surgery, The Second Hospital of Dalian Medical University, Dalian, China

## Abstract

Long non-coding RNAs (lncRNAs), which are important functional regulators in cancer, have received increased attention in recent years. In this study, next-generation sequencing technology was used to identify aberrantly expressed lncRNAs in follicular thyroid carcinoma (FTC). The long non-coding RNA–HLA complex P5 (HCP5) was found to be overexpressed in FTC. The results of the qPCR analysis were consistent with the sequencing results. In addition, functional experiments showed that overexpression of HCP5 can promote the proliferation, migration, invasiveness and angiogenic ability of FTC cells. Furthermore, according to the sequencing results, HCP5 and alpha-2, 6-sialyltransferase 2 (ST6GAL2) were co-expressed in FTC. We hypothesised that ST6GAL2 may be regulated by HCP5, which would in turn mediate the activity of FTC cells. Through qPCR, immunostaining analyses and functional experiments, we determined that the expression of HCP5 was elevated and was correlated with the levels of ST6GAL2 in FTC tissues and cells. Mechanistic experiments showed that HCP5 functions as a competing endogenous RNA (ceRNA) and acts as a sponge for miR-22-3p, miR-186-5p and miR-216a-5p, which activates ST6GAL2. In summary, our study revealed that HCP5 is a tumour regulator in the development of FTC and that it may contribute to improvement of FTC diagnosis and therapy.

## Introduction

Thyroid cancer is the most common endocrine malignancy and has had a steadily increasing incidence, which indicates that it has become an important worldwide health concern over the past 30 years^1,2^. As it accounts for approximately 10–15% of thyroid cancers, follicular thyroid cancer (FTC) is difficult to diagnose via differential diagnosis, which can lead to overtreatment. Molecular diagnosis and individualised treatment therapies will be the trend of future treatment^3^.

Increasing evidence has shown that non-coding RNAs (ncRNAs) are crucial regulators in many diseases, including cancer^4^. Long non-coding RNAs (lncRNAs) are important members of the ncRNA family, are greater than 200 bases in length, and play vital roles in cancer progression^5^, including that of thyroid cancer. Several lncRNAs, such as H19 and HOTAIR, are overexpressed and promote cell proliferation and invasiveness in thyroid cancer^6,7^. In previous studies, the lncRNA–HLA complex P5 (HCP5) has been regarded as a novel genetic locus in clinical thyroid disease^8^. However, HCP5 has not been reported to be a regulatory gene associated with FTC progression.

Furthermore, microRNAs (miRNAs) are a class of ncRNAs (approximately 22 nucleotides long)^9^ that have emerged as key post-transcriptional regulators of tumourigenesis^10,11^. For instance, miR-191, miR-1 and miR-146a can regulate cells and are differentially expressed in normal and thyroid tumour tissues^12–14^. In previous studies, protein synthesis was shown to be modulated by miRNAs that are able to bind to the 3′-untranslated region of target mRNAs^15^. According to our previous research, miR146a/b promotes FTC cell proliferation via the inhibition of ST8SIA4;^16^ moreover, miR-4299 regulates FTC cell invasiveness by targeting ST6GALNAC4^17^. In addition, increasing experimental evidence suggests that lncRNAs functionally target miRNAs, which can be sponged by lncRNAs. This indicates that their function is modulated by a direct interaction with proteins^18^. Competitive endogenous RNA (ceRNA) was first reported by Leonardo Salmena et al. They proposed ceRNAs as endogenous sponges that can affect the distribution of miRNAs on their targets, thereby imposing another novel layer of posttranscriptional regulation^19^. Changes in ceRNA regulation may affect the expression of oncogenes or tumour suppressors, and therefore, they contribute to the initiation and progression of cancers^20^, which may expand our comprehension of the roles of the transcriptome and enrich our knowledge of cancer pathogenesis, diagnosis, and therapy. For example, lncRNA NEAT1 promotes malignant progression of thyroid cancer via the regulation of miRNA-214^21^. Currently, no lncRNAs that act as ceRNAs have been reported in FTC.

In this study, we reported that HCP5 was overexpressed in tumour tissue and that this lncRNA promoted proliferation, migration, invasiveness and angiogenic ability of FTC cells as well as tumour growth in vivo. Furthermore, mechanistic investigations showed that HCP5 functioned as a ceRNA by sponging miR-22-3p, miR-186-5p and miR-216a-5p, which then activated alpha-2, 6-sialyltransferase 2 (ST6GAL2). Our findings provide new insights into the molecular function of HCP5 and suggest the potential for HCP5 to be used for improvements in FTC diagnosis and therapy.

## Results

### HCP5 and ST6GAL2 expression is up-regulated in FTC tissues and cell lines

**Fig. 1:**
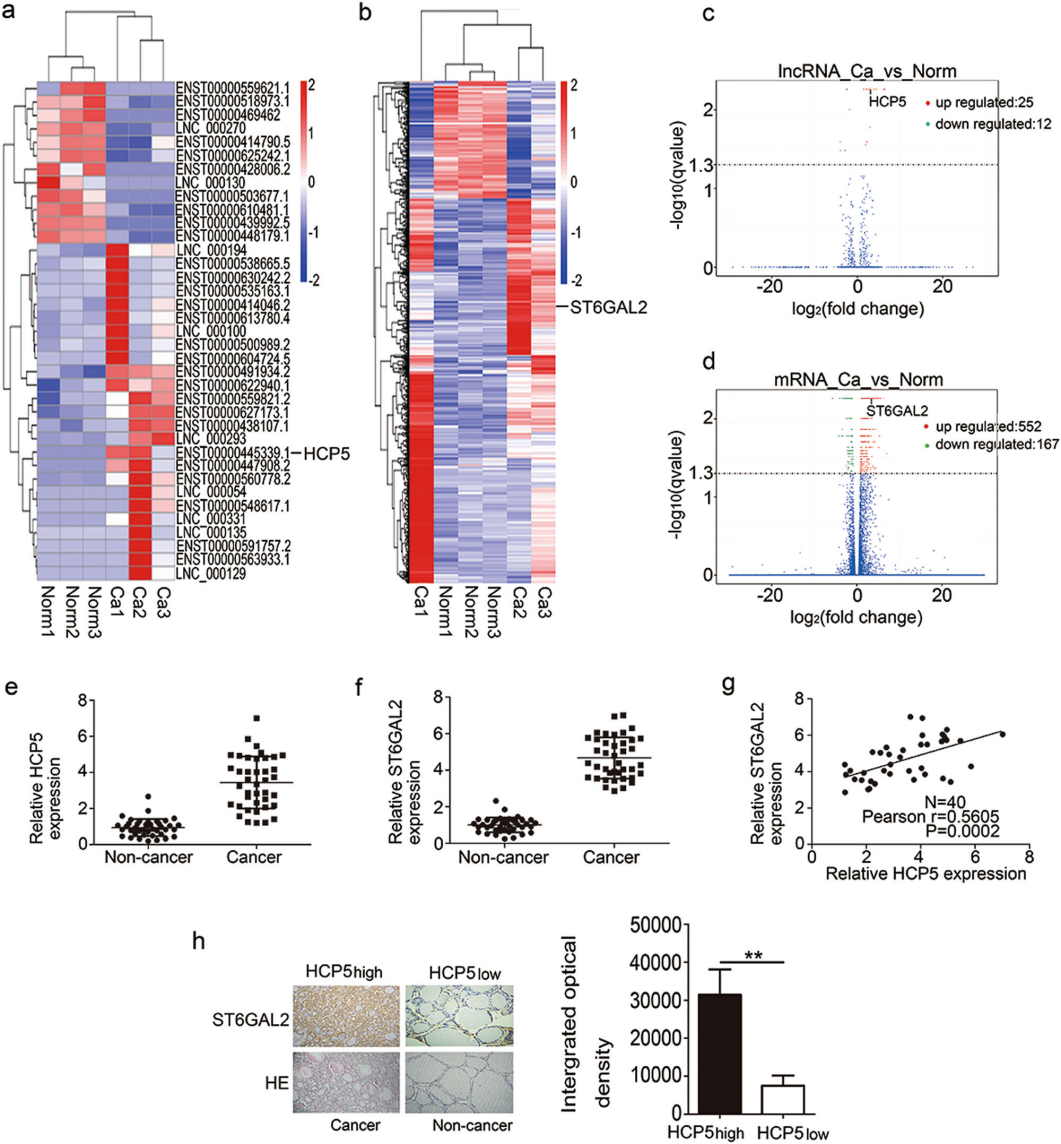
HCP5 or ST6GAL2 expression is up-regulated in FTC. **a**, **b**, **c**, **d** The heat maps and volcano plot showed that HCP5 or ST6GAL2 expression was up-regulated in FTC tissues. **e**, **f** According to the qPCR results, HCP5 and ST6GAL2 were overexpressed and **g** we found a positive relationship between ST6GAL2 and HCP5 expression in FTC tissues. **h** Immunostaining demonstrated that ST6GAL2 expression was high in the tissues with HCP5 overexpression (**pvalue < 0.01), scale bars: 20 μm

To identify the lncRNA profile and to investigate the role of lncRNAs in the development of FTC, we performed next-generation sequencing and compared the lncRNA expression in FTC patient samples with that in normal samples. The analysis of the sequencing data revealed significant changes in the lncRNA expression profile in FTC patient samples compared with normal samples. Based on *p < *0.05, *q < *0.05 and the fold change, we generated heat maps and a volcano plot. Based on these criteria, the heat maps and the volcano plot showed that 25 lncRNAs exhibited increased expression and that 12 lncRNAs exhibited decreased expression in advanced FTC (Table 1) (Fig. 1a,c). A fold change greater than or equal to 2 is considered a significant change, and among these lncRNAs, HCP5 has been reported to be a novel genetic locus in clinical thyroid disease, including when it is co-expressed with ST6GAL2. Therefore, we concentrated our efforts on HCP5 (fold change = 2.12903, *p*value = 0.00005, *q*value = 0.00548452). In terms of mRNAs, ST6GAL2 is a member of the sialyltransferase (ST) family, which has been the focus of much of our research. Among the 552 mRNAs examined, ST6GAL2 (fold change = 2.29387, *p*value = 0.00005, *q*value = 0.005485) was dramatically up-regulated in advanced FTC (Fig. 1b,d). Next, we examined HCP5 and ST6GAL2 expression by qPCR in 40 pairs of tissues from FTC patients. According to the qPCR results, HCP5 and ST6GAL2 were overexpressed in FTC tissues (Fig. 1e,f). The qPCR results were consistent with the sequencing results. Through data analysis, we found a positive relationship between ST6GAL2 expression and HCP5 in FTC tissues (Fig. 1g). Correspondingly, immunostaining demonstrated that ST6GAL2 expression was high in the tissues that overexpressed HCP5 (Fig. 1h).

**Table 1:** Top 10 lncRNAs in FTC

Gene id	Gene name	Ca FPKM	Norm FPKM	log2 (fold change)	*p*value	*q*value
XLOC_024351	–	118.311	1.40605	6.3948	5.00E-05	0.00548
ENSG00000206339.10	HCP5	20.5134	4.6896	2.12903	5.00E-05	0.00548
ENSG00000259417.2	LINC01314	5.24405	0.23715	4.46681	5.00E-05	0.00548
ENSG00000269926.1	RP11-442H21.2	43.418	9.51732	2.18967	0.00035	0.02557
ENSG00000281852.1	LINC00891	5.25587	1.87458	1.48737	5.00E-05	0.00548
XLOC_048947	–	3.65186	0.585012	2.64209	5.00E-05	0.00548
ENSG00000259459.5	RP11-321G12.1	9.19891	1.08677	3.08142	5.00E-05	0.00548
ENSG00000225783.6	MIAT	3.84391	0.935367	2.03897	5.00E-05	0.00548
ENSG00000255733.5	IFNG-AS1	3.52673	0.235901	3.90208	5.00E-05	0.00548
ENSG00000234449.2	RP11-706O15.3	4.93676	0.743829	2.73052	5.00E-05	0.00548

**Fig. 2:**
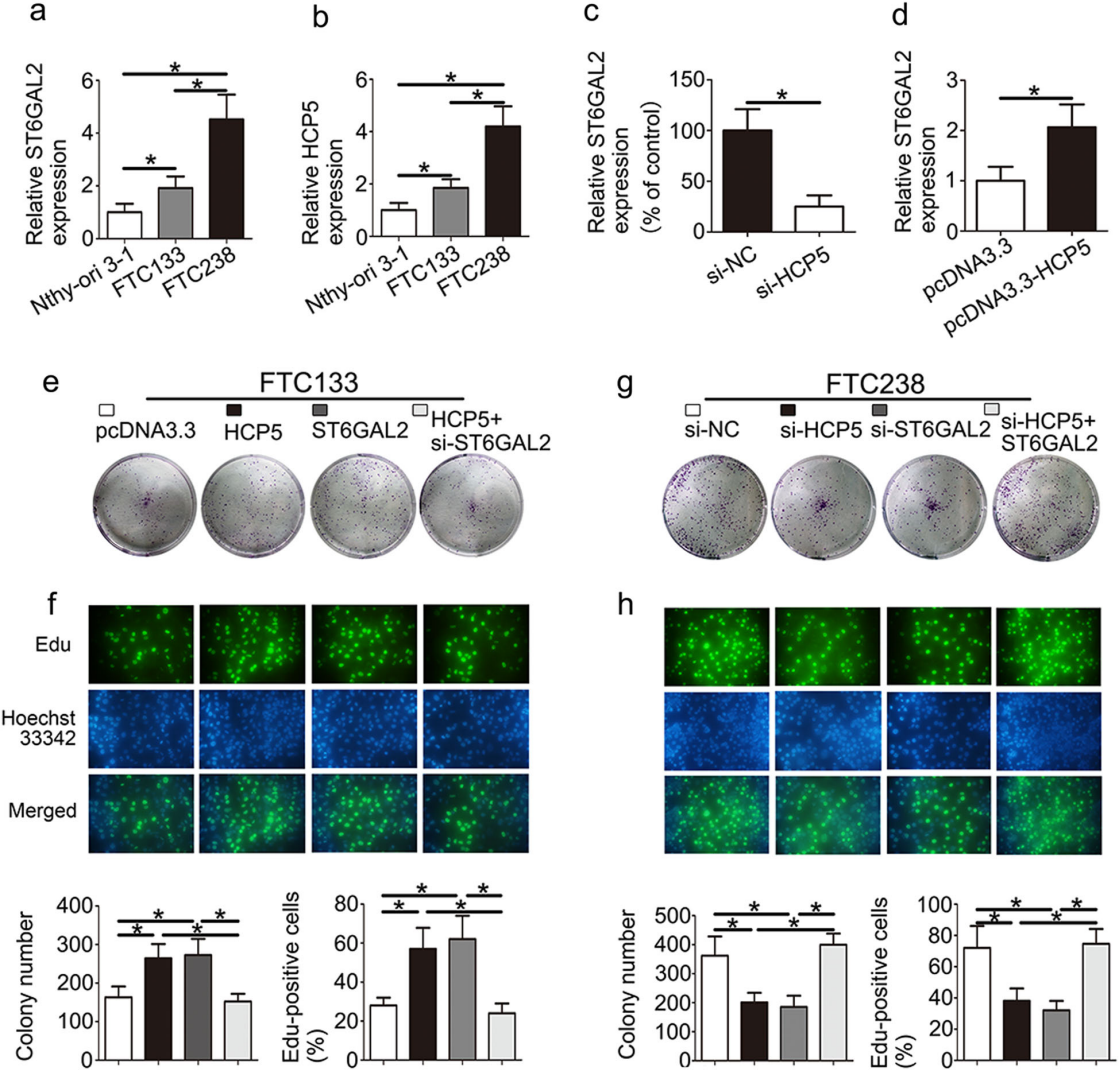
HCP5 and ST6GAL2 regulate the proliferation of FTC cells. **a**, **b** High expression of HCP5 and ST6GAL2 was observed in FTC238 cells. **c**, **d** The expression of ST6GAL2 was down-regulated in the si-HCP5 group and up-regulated in the HCP5 group. HCP5 and ST6GAL2 promoted the proliferation of FTC cells according to **e** a colony formation assay and **f** an Edu assay in FTC133 cells. **g,** **h** In contrast, the inhibition of HCP5 and ST6GAL2 could suppress the proliferation of FTC238 cells. (*pvalue < 0.05), scale bars: 20 μm

Furthermore, the expression of HCP5 and ST6GAL2 was increased in FTC238 (highly invasive) cells compared with FTC133 (poorly invasive) and Nthy-ori 3-1 cells (Fig. 2a,b). To speculate as to the relationship between HCP5 and ST6GAL2 in vivo, the expression of ST6GAL2 was down-regulated in the si-HCP5 group and was up-regulated in the HCP5 group (Fig. 2c,d). As a result, HCP5 and ST6GAL2 were overexpressed in FTC tissues and cell lines. Moreover, a western blot analysis also demonstrated similar results (Supplementary 1a and b).

### HCP5 and ST6GAL2 regulate cell proliferation, migration, invasiveness and angiogenic ability of FTC cells

**Fig. 3:**
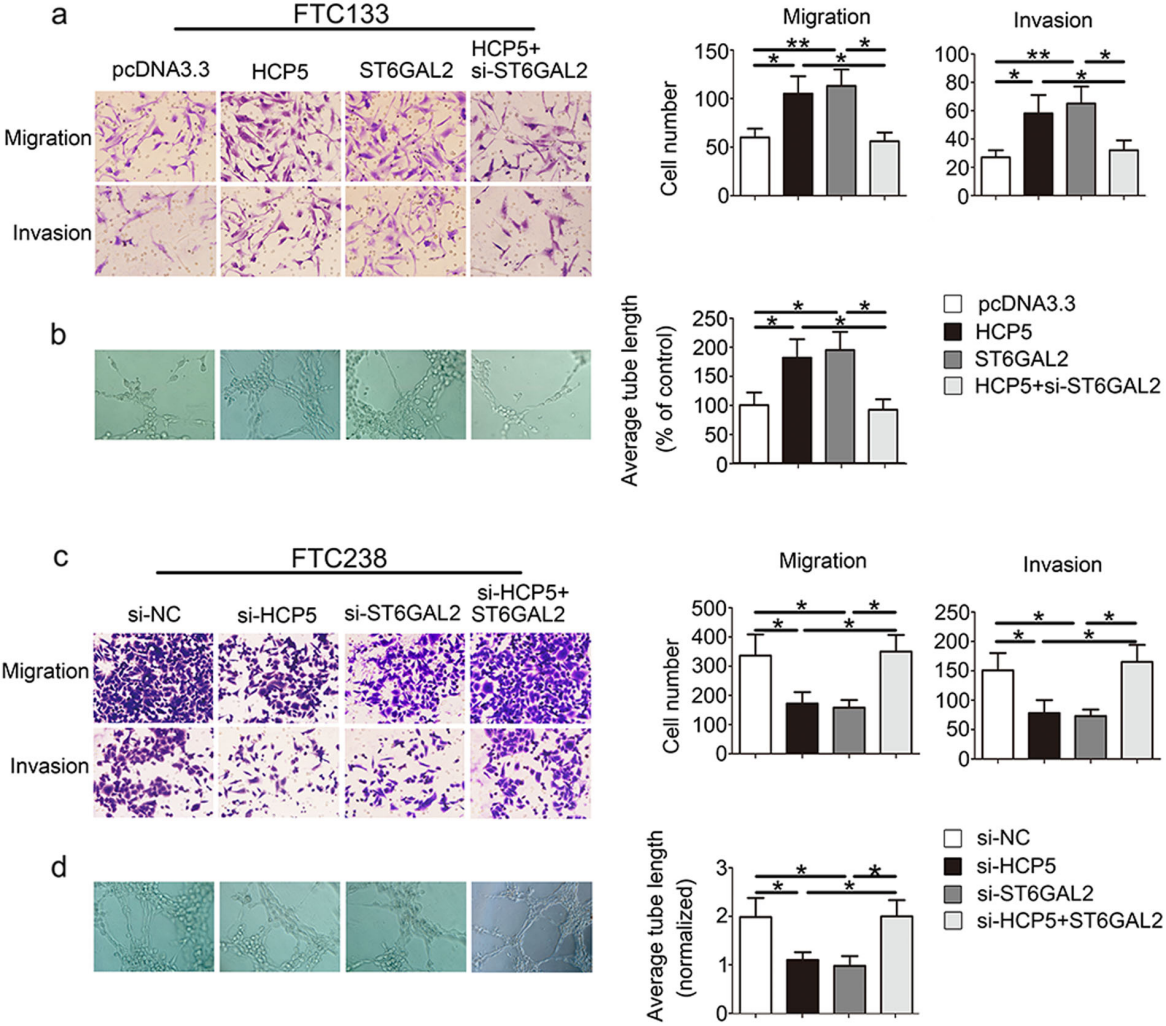
HCP5 regulates the migration, invasiveness and angiogenic ability of FTC cells. HCP5 can promote the development of FTC via enhancements in **a** migration, invasiveness and **b** angiogenic capacity of FTC cells, but **c**, **d** opposite results were found in the si-HCP5 and si-ST6GAL2 groups. (**pvalue < 0.01, *pvalue < 0.05), scale bars: 20 μm

We investigated the role of HCP5 and ST6GAL2 in proliferation using a colony formation assay, an Edu assay and a CCK8 assay. The colony formation assay showed that overexpression of HCP5 and ST6GAL2 promoted cell proliferation, while the inhibition of HCP5 or ST6GAL2 reduced cell proliferation (Fig. 2e, g). The result of the Edu (Fig. 2f,h) and CCK8 assays (Supplementary 1c and d) showed similar results with respect to proliferation. Next, we evaluated cancer cell migration and invasion using Transwell-based assays. As shown in Fig. 3a, cell migration and invasion were up-regulated in the presence of HCP5 and ST6GAL2, while the opposite result was observed in which HCP5 or ST6GAL2 was inhibited (Fig. 3c). A tube formation assay was used to assess the effect of HCP5 on tumour development. The length of tubes was increased by proangiogenic stimuli from the supernatant of the HCP5 and ST6GAL2 groups (Fig. 3b); the opposite results were found in the si-HCP5 and si-ST6GAL2 groups (Fig. 3d). Moreover, the ability of FTC cells to be regulated by HCP5 was rescued by the reintroduction of ST6GAL2, while si-ST6GAL2 could attenuate the effect of HCP5. These data suggest that HCP5 and ST6GAL2 are able to promote the proliferation, migration, invasiveness and angiogenic ability of FTC cells.

### HCP5 promotes FTC cell proliferation in vivo

**Fig. 4:**
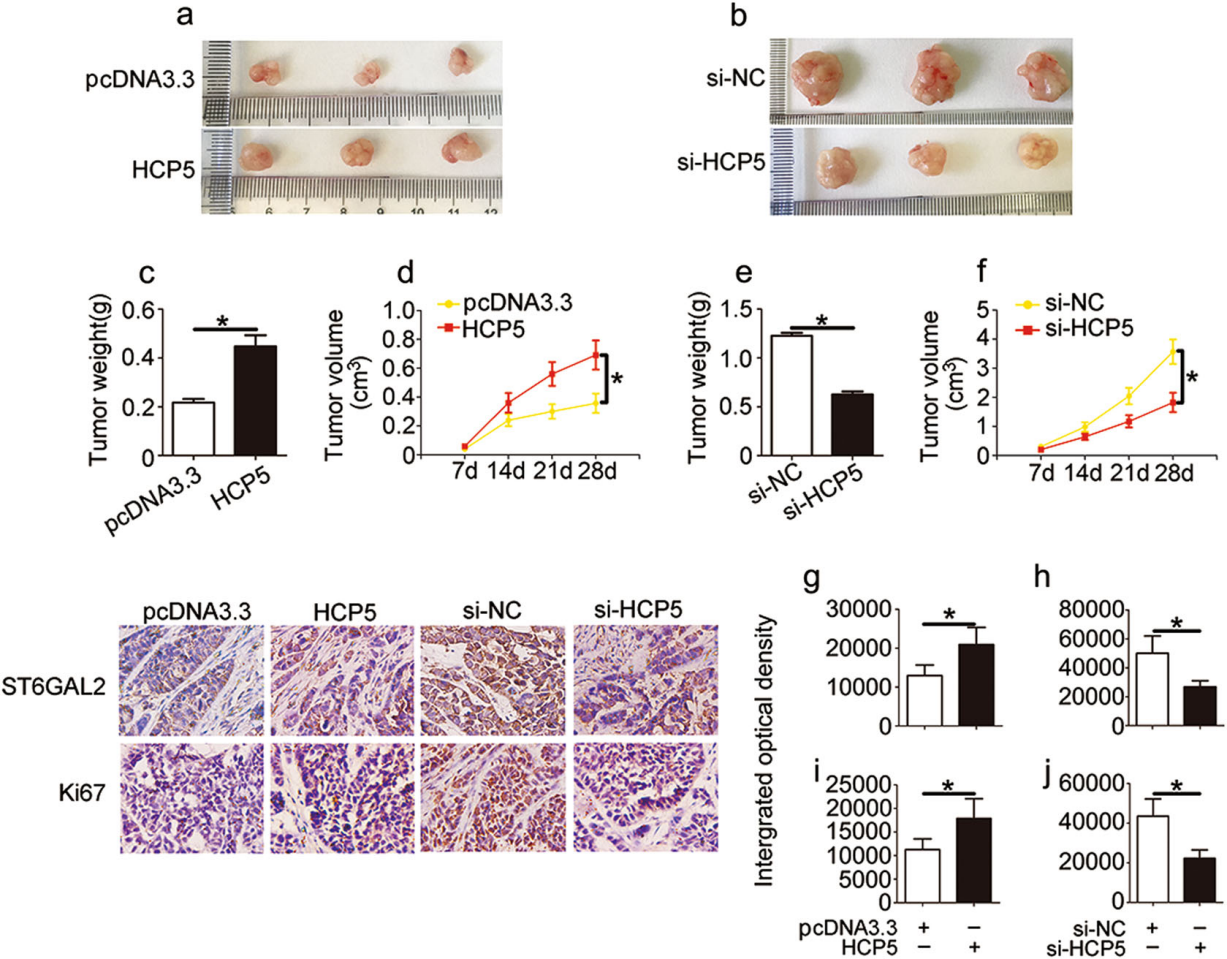
HCP5 regulates FTC cell growth in vivo. **a**, **d** Tumour growth curves were measured after injection of FTC133 cells transfected with HCP5 (**b**, **f**) and FTC238 cells transfected with si-HCP5. **c**, **e** Tumour weights were measured after the tumours were removed. Immunofluorescence staining using antibodies against **g**, **i** ST6GAL2 and **h**, **j** Ki67 was used to assess proliferation capacity. (*pvalue < 0.05), scale bars: 20 μm

We investigated the in vivo activity of HCP5 in nude mice. To confirm whether HCP5 affects tumourigenesis, we examined FTC133 cells transfected with pcDNA3.3 and HCP5 as well as FTC238 cells transfected with si-NC and si-HCP5. The transfection efficiency was approximately 82%. After 4 weeks, tumour growth was more rapid (Fig. 4a), and tumour weight and volume were dramatically increased in the HCP5 group (Fig. 4c, d). In contrast, tumour growth, indicated by weight and volume, was significantly decreased in the si-HCP5 group (Fig. 4b, e, f). To quantify the level of HCP5, tumours were excised from the nude mice and used for the qPCR analysis. We found that the level of HCP5 was increased in the HCP5/FTC133 group and was reduced in the si-HCP5/FTC238 group (Supplementary 1e and f). Correspondingly, an immunostaining analysis of xenografted tumour tissues revealed that ST6GAL2 and Ki67 expression was also increased in the FTC133/HCP5 group (Fig. 4g, i), whereas the inverse was found in the FTC238/si-HCP5 group (Fig. 4h, j). Taken together, these data suggest that HCP5 is involved in tumourigenesis and that it promotes FTC cell proliferation in vivo.

### HCP5 functions through the ceRNA sponging pattern of ***miR-22-3p, miR-186-5p and miR-216a-5p***

**Fig. 5:**
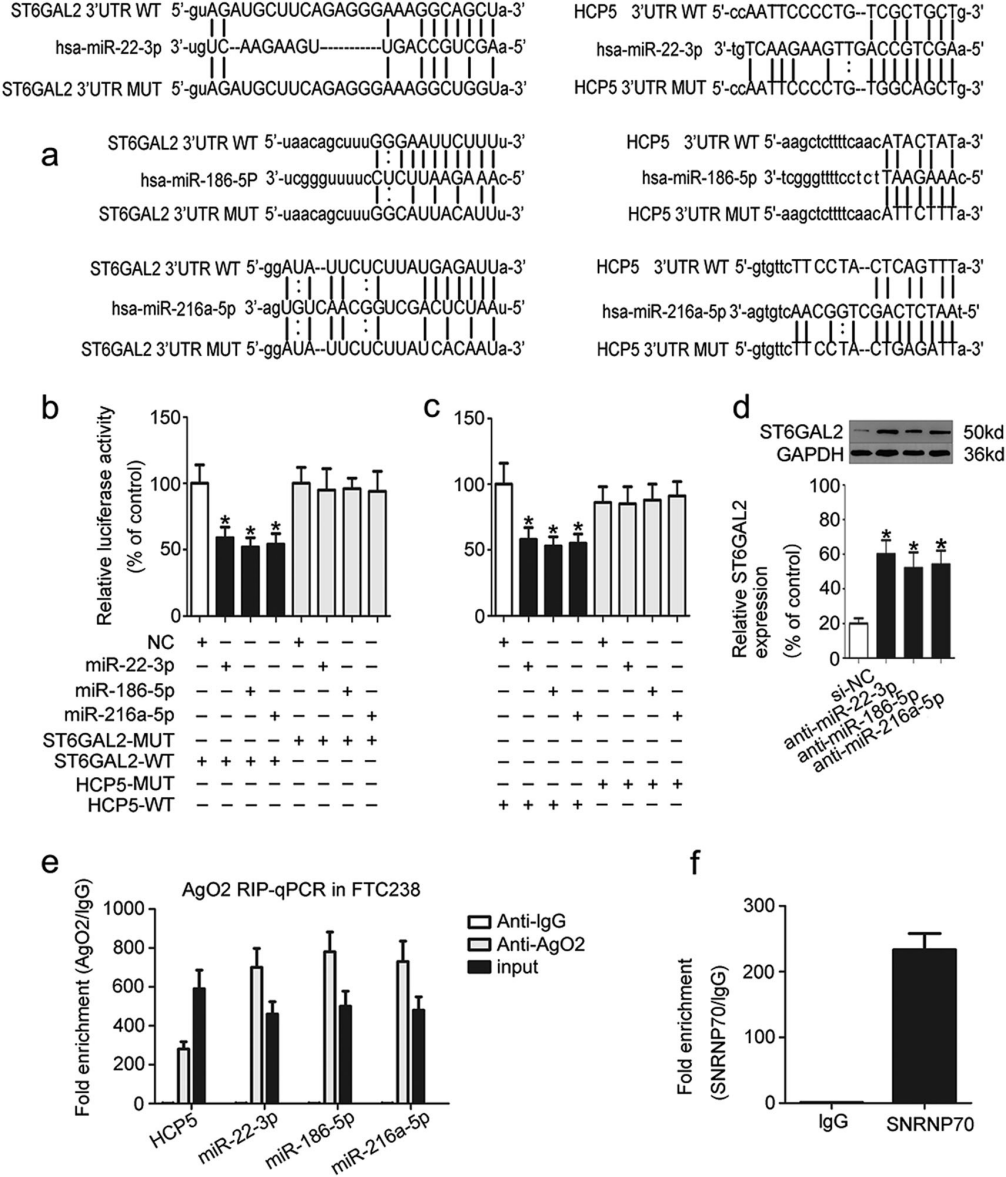
HCP5 functions as a ceRNA and sponges miR-22-3p, miR-186-5p and miR-216a-5p. **a** Using two programs (starbase and microRNA.org), we found that both HCP5 and ST6GAL2 are potential targets of miR-22-3p, miR-186-5p and miR-216a-5p. **b**, **c** A dual luciferase reporter assay was used to test whether HCP5 and ST6GAL2 are targets of miR-22-3p, miR-186-5p and miR-216a-5p. **d** Western blot analysis showed that the knockdown of miR-22-3p, miR-186-5p, or miR-216a-5p in FTC133 cells triggered a significant increase in ST6GAL2 protein expression. **e**, **f** An RIP experiment showed that HCP5 was enriched in Ago2-contaning miRNAs compared with IgG; miR-22-3p, miR-186-5p and miR-216a-5p were also detected in the same precipitate (*pvalue < 0.05)

To further study the mechanism of HCP5, we used two programmes, starbase and microRNA.org, and found that both HCP5 and ST6GAL2 are potential targets of miR-22-3p, miR-186-5p and miR-216a-5p (Fig. 5a). Next, a dual-luciferase reporter assay was used to test whether HCP5 and ST6GAL2 are targets of miR-22-3p, miR-186-5p and miR-216a-5p. The luciferase assay showed that the luciferase activity was reduced in HEK293T cells that were co-transfected with miR-22-3p, miR-186-5p, miR-216a-5p and ST6GAL2-WT or HCP5-WT but was not reduced in HEK293T cells containing ST6GAL2-MUT (Fig. 5b) or HCP5-MUT (Fig. 5c). The luciferase assay was performed as previously described^22^, and miRNA-125a-3p/FUT6 was used as a positive control for the luciferase assay (Supplementary 1h). These results confirm that miR-22-3p, miR-186-5p and miR-216a-5p target ST6GAL2 and HCP5. A western blot analysis showed that the knock down of miR-22-3p, miR-186-5p or miR-216a-5p in FTC133 cells triggered a significant increase in ST6GAL2 protein expression (Fig. 5d). Furthermore, it is well known that miRNA is present in the cytoplasm as a component of the RNA-induced silencing complex (RISC), which also contains Ago2. Ago2 is the key component of the RISC and is necessary for miRNA-mediated gene silencing. An RNA immunoprecipitation (RIP) experiment was performed in FTC238 cell extracts using antibodies against Ago2 to determine whether HCP5 and miR-22-3p, miR-186-5p or miR-216a-5p are part of the same RISC. The RIP results revealed that HCP5 was enriched in Ago2-contaning miRNAs compared with control immunoglobulin G (IgG) immunoprecipitates. miR-22-3p, miR-186-5p and miR-216a-5p were also detected in the same precipitate (Fig. 5e). Next, SNRNP70 was used as a positive control for the RIP procedure (Fig. 5f). Pull-down assays with an HCP5 biotinylated probe were then performed to verify their direct binding. Compared with the negative control, miR-22-3p, miR-186-5p and miR-216a-5p were pulled down and detected in the HCP5 biotinylated probed RNA–RNA complexes by qPCR (Supplementary 1g). These results suggest that HCP5 regulates FTC cells by targeting ST6GAL2 according to the ceRNA patterns (Fig. 7).

### HCP5 activity is partially activated through negative regulation of ***miR-22-3p, miR-186-5p or miR-216a-5p***

**Fig. 6:**
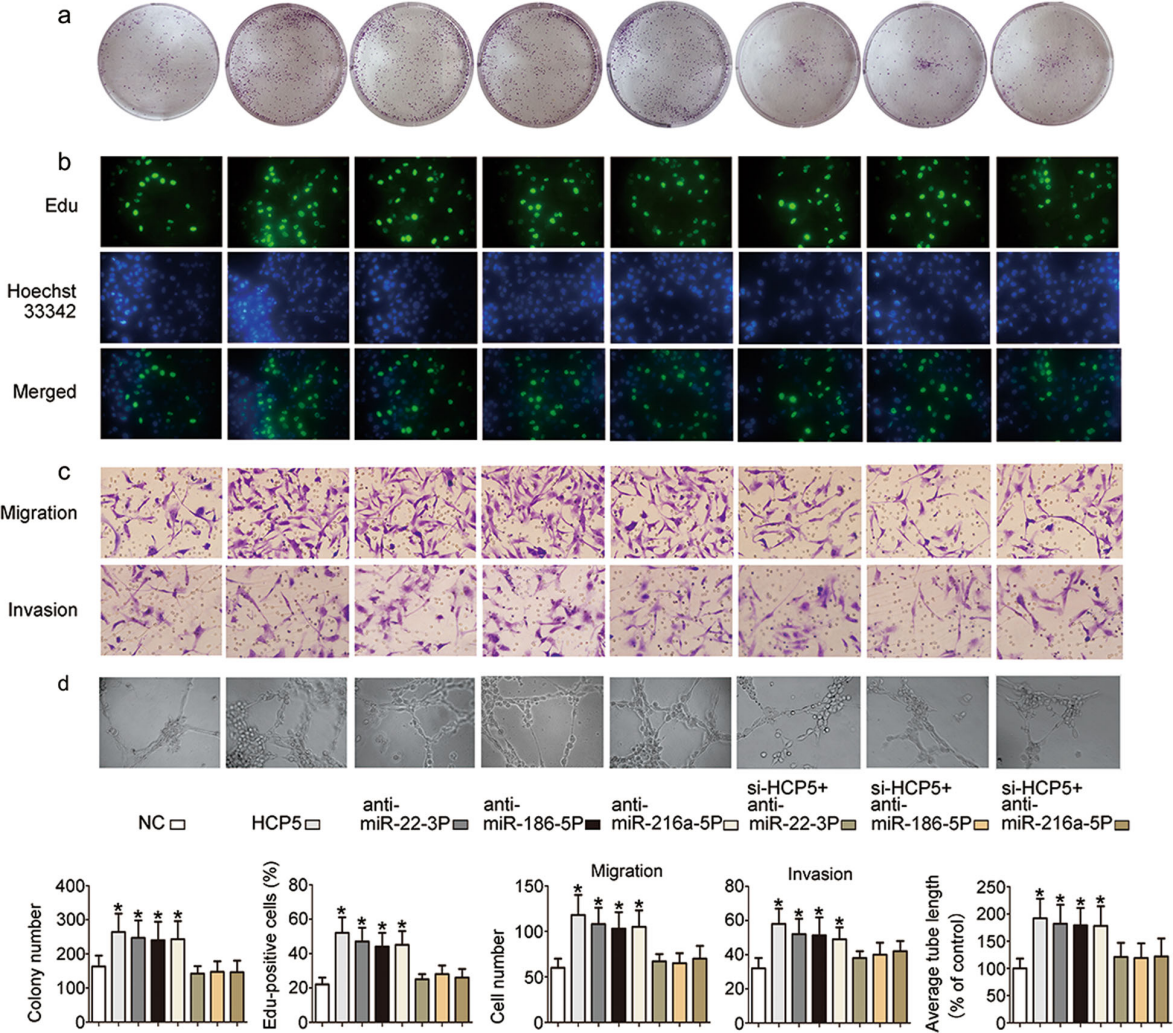
The function of HCP5 is partially dependent on miR-22-3p, miR-186-5p and miR-216a-5p inhibition. The effects of si-miR-22-3p, si-miR-186-5p and si-miR-216a-5p on **a** and **b** cell proliferation, **c** migration, invasiveness and **d** angiogenesis were attenuated by si-HCP5. (*pvalue < 0.05), scale bars: 20 μm

To study the function of miR-22-3p, miR-186-5p and miR-216a-5p, functional experiments were performed using FTC133 cells. The results showed that the proliferation (Fig. 6a,b), migration, invasiveness (Fig. 6c) and angiogenic ability (Fig. 6d) of FTC133 cells were up-regulated in the HCP5, si*-*miR-22-3p, si-miR-186-5p and si-miR-216a-5p groups. These properties in FTC cells were attenuated by si-HCP5. As expected, these data indicate that the function of HCP5 is partially dependent on miR-22-3p, miR-186-5p and miR-216a-5p.

**Fig. 7:**
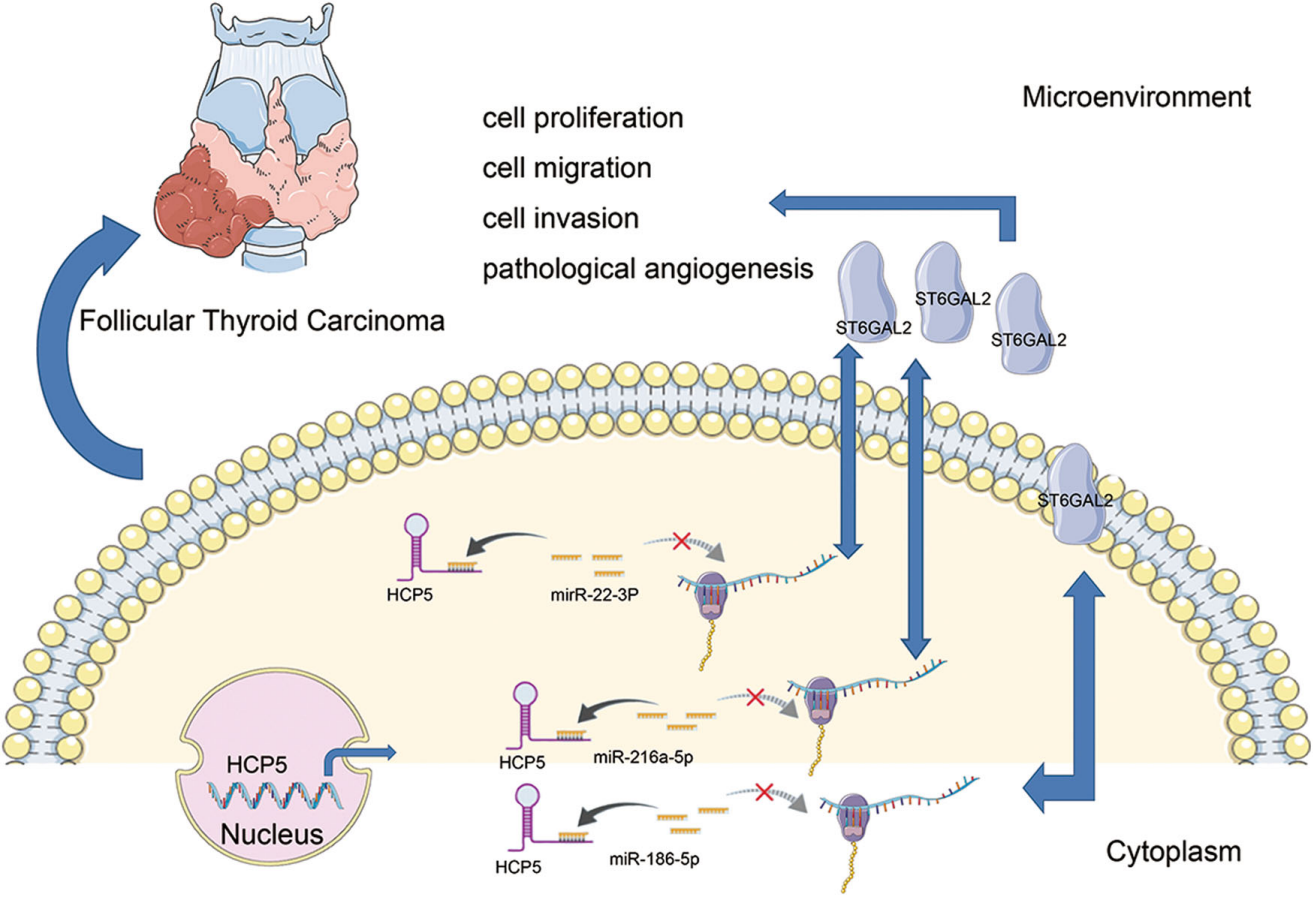
Schematic representations of pathways modulated by HCP5 in FTC. HCP5 is up-regulated in FTC and promotes FTC progression via a ceRNA pattern by sponging miR-22-3p, miR-186-5p and miR-216a-5p, which activate ST6GAL2

## Discussion

Follicular thyroid carcinoma and follicular thyroid adenoma (FTA) are associated with follicular cell differentiation^23^. It is difficult to distinguish FTC from FTA based on cytologic, sonographic or clinical features alone^24^. Although ultrasound is the best imaging modality for the detection of thyroid tumours, this method cannot accurately distinguish FTC from FTA^25^. Furthermore, treatment planning and prognosis of patients with FTC largely depend on a correct tumour diagnosis. Currently, patients with FTA are almost always treated with partial/hemithyroidectomy, while patients with FTC are treated with total thyroidectomy and are recommended to take thyroxine tablets for life. Therefore, the diagnosis and therapy of FTC need improvement. In recent years, knowledge regarding the molecular alterations that underlie thyroid cancer has increased, which has greatly augmented interest in the discovery of new molecular markers for diagnosis and treatment^26^.

LncRNAs, as important non-coding RNAs, have strong organismal complexity and are found in every branch of life^27^. Recent papers have revealed that lncRNA expression profiles are important markers for diverse human cancers^28,29^. For example, HULC is the first lncRNA that has been shown to be specifically up-regulated in hepatocellular carcinoma (HCC), and it can also be used as a biomarker^30^. HOTAIR plays important roles in breast cancer and may be an important target for diagnosis and therapy^31^. Our study investigated the potential involvement of lncRNA-mediated mechanisms in FTC. To our knowledge, this is the first study to systematically investigate the expression profile of lncRNAs in FTC. We investigated lncRNA expression profiles by next-generation sequencing and identified HCP5 as the significative lncRNA in advanced FTC. In previous studies, HCP5 was shown to be a susceptibility locus for HCV-related HCC^32^. In clinical thyroid disease, HCP5 is regarded as a novel genetic locus^8^. However, the biological consequences of HCP5 dysregulation in FTC progression have not yet been reported.

In this study, we analysed HCP5 expression in FTC by qPCR. The results showed that HCP5 was significantly up-regulated in FTC tissues, which is consistent with the sequencing results. We speculated that up-regulation of HCP5 may promote an aggressive tumour phenotype. Our data from the functional study confirmed that HCP5 promotes FTC progression by enhancing the proliferation, migration, invasiveness and angiogenic capacity of FTC cells as well as tumour growth in vivo. The results suggest that HCP5 participates in a potential pathway that functions in FTC progression and that it may be a promising prognostic biomarker for FTC patients. Furthermore, lncRNAs act as crucial regulators of gene expression via the regulation of cancer proliferation and invasion^4^. Therefore, we further investigated the expression of various mRNAs. According to the next-generation sequencing data, among the 552 mRNAs examined, ST6GAL2 was dramatically up-regulated in advanced FTC. Due to our lack of continued awareness of other genes, we concentrated our efforts on ST6GAL2, which is a member of the ST family.

STs are glycosyltransferases that control the linkage and level of cell surface sialic acids, and as such, they play key roles in many biological processes of human disease, including cancer^33^. STs are categorised into four families (ST6GAL, ST3GAL, ST6GALNAC and ST8SIA)^34,35^. Previous studies have reported that ST6GALNAC2 acts as a suppressor of breast cancer metastasis^36^ and that it can also be used for early detection of human colorectal carcinomas^37^. According to our previous reports, ST6GAL1 and ST8SIA2 regulate chemosensitivity of HCC^38^, and ST3GAL6 mediates cell growth and invasion in HCC^39^. ST6GALNAC2 regulates breast cancer invasion^40,41^. In FTC research, we found that ST6GALNAC4 and ST6GALNAC2 modulate tumourigenicity of FTC^17,42^ and that miR146a/b promotes FTC cell proliferation via the inhibition of ST8SIA4^16^. In the current study, ST6GAL2 was more highly expressed in FTC tissues and cell lines compared with normal tissues and cell lines. Through data analysis, we found a positive relationship between ST6GAL2 and HCP5 expression. According to functional analyses, we found that the over-expression of ST6GAL2 promoted cellular activities in FTC cells. Moreover, immunostaining demonstrated that the expression of ST6GAL2 was up-regulated in the FTC tissues with high expression of HCP5 as well as in xenograft tumour tissues in the FTC133/HCP5 group. According to these results, we speculate that HCP5 promotes FTC cell ability via the regulation of ST6GAL2.

Recent publications have revealed that lncRNAs are important and powerful regulators of gene activity^43^. One factor related to lncRNAs that has received considerable attention is the ceRNA hypothesis^44^. For example, lncRNA H19 functions as a ceRNA by regulating the expression of miR-17-5p in thyroid cancer^7^, and lncRNA NEAT1 promotes the malignant progression of thyroid cancer by regulating miRNA-214^21^. In this study, we speculated that HCP5 functions as a ceRNA by sponging miRNAs, which leads to ST6GAL2 activation. To explore the underlying mechanism of HCP5, starbase was used to predict its miRNA targets. We found that both HCP5 and ST6GAL2 are potential targets of miR-22-3p, miR-186-5p and miR-216a-5p. A luciferase assay was used to confirme that miR-22-3p, miR-186-5p and miR-216a-5p target both ST6GAL2 and HCP5. Moreover, through use of antibodies against Ago2, RIP showed that HCP5 and miR-22-3p, miR-186-5p or miR-216a-5p are part of the same RISC. Furthermore, our data from a functional study indicated that the function of HCP5 is partially dependent on the inhibition of miR-22-3p, miR-186-5p and miR-216a-5p. These data are consistent with the hypothesis that HCP5 functions as a ceRNA by sponging miR-22-3p, miR-186-5p and miR-216a-5p, which activate ST6GAL2. According to a previous report, the prospect of using ceRNAs in cancer therapy is very promising^45^, and the effectiveness of a ceRNA depends on the number of microRNAs that it can “sponge”^19^. For the first time, we identified that HCP5 acts as a powerful regulator in FTC and that it functions as a ceRNA by sponging miR-22-3p, miR-186-5p and miR-216a-5p. These results highlight the role of HCP5 and demonstrate that targeting HCP5 could be a promising therapeutic strategy in FTC patients. However, we are aware of the limitation of the present study in that although we used multiple independent sets of FTC samples, larger cohorts are required to validate the association between HCP5 expression and patient survival.

In summary, we show that HCP5 is significantly up-regulated in FTC and that it promotes FTC progression. Furthermore, a mechanistic study showed that HCP5 functions as a ceRNA. Our results provide new insight into the mechanism that links the function of HCP5 with FTC and suggests that HCP5 can serve as a novel potential target for the improvement of FTC diagnosis and therapy. Future studies should explore whether HCP5 can affect BRAF mutations, which occur concomitantly in thyroid cancer.

## Materials and methods

### Tissue collection

FTC samples were obtained from 43 patients who provided informed consent, which was in accordance with the ethical standards of the Second Hospital of Dalian Medical University (Dalian, China) Review Board. All samples were reviewed by a pathologist and were histologically confirmed as FTC based on a histopathological evaluation. No local or systemic treatments administered in these patients before surgery.

### Cell lines

The FTC cell lines FTC-133 and FTC-238 were purchased from the ECACC (Shanghai, China). The thyroid cell line Nthy-ori 3-1 was purchased from Jennio Biotech Co (Guangzhou, China). The human embryonic kidney cell line HEK293T and umbilical vein endothelial cells (HUVECs) were obtained from the Institute of Biochemistry (Shanghai, China). FTC133, Nthy-ori 3-1, HEK293T cells and HUVECs were cultured in DMEM (Gibco, Grand Island, NY, USA) supplemented with 10% heat-inactivated foetal bovine serum (FBS) (Gibco). FTC-238 cells were cultured in RPMI 1640 medium (Gibco) supplemented with10% FBS. All cells were incubated at 37 °C in a humidified and 5% CO_2_ incubator.

### Next-generation sequencing technology

The next-generation sequencing work was performed by Novogene (Beijing, China). A total amount of 3 μg RNA per sample was used as input material for the RNA sample preparations. RNA degradation and contamination were monitored on 1% agarose gels. RNA purity was checked using a NanoPhotometer^®^ spectrophotometer (IMPLEN, CA, USA). RNA concentration was measured using a Qubit^®^ RNA Assay Kit in a Qubit^®^ 2.0 Fluorometer (Life Technologies, CA, USA). RNA integrity was assessed using a RNA Nano 6000 Assay Kit and a Bioanalyzer 2100 system (Agilent Technologies, CA, USA). The Ballgown suite includes functions for the interactive exploration of the transcriptome assembly, visualisation of transcript structures, an abundance of features specific for each locus, and post hoc annotation of assembled features to annotated features. Transcripts with a *p*-adjust < 0.05 were determined to be differentially expressed. The sample size consisted of three tissue samples per group.

### PCR analysis

RNA extraction from cell lines and tissues was performed using TRIzol reagent (Invitrogen, Carlsbad, CA, USA). RNA was reverse-transcribed into cDNA with the QuantiTect Reverse Transcription Kit (QIAGEN, Valencia, CA, USA). Real-time PCR analyses were quantified by SYBR-Green (Takara, Otsu, Shiga, Japan), and the levels were normalised to the level of GAPDH. The sequences of the upstream and downstream primers are as follows: HCP5, 5′-CCG CTG GTC TCT GGA CAC ATA CT-3′ and 5′-CTC ACC TGT CGT GGG ATT TTG C-3′. ST6GAL2: 5′-ACG CTG CTG ATT GAC TCT TCT-3′ and 5′-CAC ATA CTG GCA CTC ATC TAA-3′. GAPDH: 5′-CTC CTC CAC CTT TGA CGC TG-3′ and 5′-TCC TCT TGT GCT CTT GCT GG-3′.

### IHC staining analysis

Paraffin-embedded tissues were immunostained for ST6GAL2 and Ki67 proteins. The slides were dried, deparaffinized and rehydrated. The signal was amplified and visualised with DAB (Santa Cruz Biotechnology) substrate chromogen solution, followed by counterstaining with haematoxylin. Anti-ST6GAL2 (1:200) and anti-Ki67 (1:200) were purchased from Abcam (Cambridge, UK). Image-ProPlus 6.0 Software (Media Cybernetics, USA) was used for the protein expression analysis.

### RNA interference and plasmid constructs

We purchased siRNAs against HCP5 and ST6GAL2 as well as a miR-22-3p inhibitor, miR-186-5p inhibitor, miR-216a-5p inhibitor and negative control siRNA from GenePharma (Shanghai, China). Full-length HCP5-pcDNA 3.3 vectors and ST6GAL2-pcDNA 3.3 were purchased from GenePharma. The cell lines were seeded in six-well plates and were transfected with specific siRNAs and plasmid vectors using FuGENE HD Transfection Reagent (Promega, USA). The sequences of the siRNA primers are as follows: si-HCP5 (5′–3′): CAC GUG UUC UUC CUA CUG ATT and UCA GUA GGA AGA ACA CGU GTT; si-ST6GAL2 (5′-3′): GCA UCG AGU GUG UCA GUU AUA and UAA CUG ACA CAC UCG AUG CUG; miR-22-3p inhibitor: AAG CUG CCA GUU GAA GAA CUG U; miR-186-5p inhibitor: CAA AGA AUU CUC CUU UUG GGC U; miR-216a-5p inhibitor: UAA UCU CAG CUG GCA ACU GUG A. Based on the qPCR results, the transfection efficiency of si-HCP5, si-ST6GAL2, the miR-22-3p inhibitor, the miR-186-5p inhibitor and the miR-216a-5p inhibitor was approximately 82, 83, 85, 86 and 85%, respectively.

### Ethynyldeoxyuridine (Edu) analysis

The cells were cultured in 96-well plates at a density of 4 × 10^4^ cells/well. Forty-eight hours after transfection, 20 μM Edu labelling media (KeyGENBioTECH, Nanjing, China) was added to the 96-well plates, which were then incubated for 2 h at 37 °C and 5% CO_2_. After treatment with 4% paraformaldehyde and 0.5% Triton X-100, the cells were stained with anti-Edu working solution. The percentage of Edu-positive cells was calculated after fluorescence microscopy analysis.

### Colony formation assay

With respect to the colony formation assay, 1 × 10^3^ cells were seeded in six-well plates. The cells were mixed and then cultured for 1 week in culture medium with 10% FBS. Clusters containing ≥30 cells were counted as a single colony.

### Cell migration and invasion assays

The cells were harvested, resuspended in serum-free media and placed into the upper chamber of a Transwell membrane filter (Corning, NY, USA) for the migration assays or in the upper chamber of a Transwell membrane filter coated with Matrigel (Corning) for the invasion assays. Culture medium with 10% FBS was added to the lower compartment of the chamber and was used as a chemoattractant. After 24 h of incubation, the cells were stained with methanol and 0.1% crystal violet, imaged, and counted using an Olympus microscope (Tokyo, Japan).

### Endothelial tube formation assay

Matrigel (Corning) (50 μL) was added to each well of a 96-well plate and was allowed to solidify (at 37 °C for 30 min). HUVECs were resuspended in supernatant collected from the RNAi, RNA and control groups. Then, 300 μL of supernatant was added to each well containing 4 × 10^4^ HUVECs, which were incubated at 37 °C. After 8 h, tube formation was observed under a microscope.

### In vivo tumourigenicity assay

All animal experiments were performed according to the guidelines of the responsible governmental animal ethics committee. Nude mice (6 weeks old) were obtained from the Animal Facility of Dalian Medical University. To establish BC xenografts, approximately 1 × 10^6^ tumour cells in 0.1 mL of phosphate-buffered saline were injected subcutaneously into the right flank of the nude mice. Once palpable tumours were observed (after approximately 4 weeks), the animals were euthanized, and the tumours were excised.

### Western blot assay and antibodies

Western blot analysis was performed as previously described (22). An antibody against ST6GAL2 (ab69276, Abcam, Cambridge, UK, 1:1000) was used.

### RNA Binding Protein Immunoprecipitation (RIP) assay

RIP was performed according to the protocol of EZMagna RIP kit (Millipore, Billerica, MA, USA). FTC238 cells at 80–90% confluency were scraped off and lysed in complete RIP lysis buffer. Then, 100 μL of whole cell extract was incubated with RIP buffer containing magnetic beads conjugated to a human anti-Ago2 antibody (Millipore), negative control normal mouse IgG (Millipore) or anti-SNRNP70 (Millipore), which was used as positive control for the RIP procedure. The samples were incubated with Proteinase K (30 min at 55 °C) to remove the protein. The RNA concentration was measured using a NanoDrop (Thermo Scientific, Beijing), and the RNA quality was assessed using a bioanalyser (Agilent, Santa Clara, CA, USA). Furthermore, the immunoprecipitated RNA was purified and analysed by qRT-PCR.

### Dual-luciferase reporter assays

Dual-luciferase reporter assays were performed in HEK293T cells as previously described^22^. Briefly, miR-22-3p, miR-186-5p or miR-216a-5p and wild-type or mutants of ST6GAL2 or the HCP5 fragment were co-transfected into HEK293T cells along with 2 ng of pRL-TK (Promega) using FuGENE HD Transfection Reagent (Promega) Luciferase activity was detected using the Dual-Luciferase Reporter Assay System (Promega) according to the manufacturer’s instructions, and the ratio of Firefly to Renilla luciferase activity was determined.

### Statistical Analysis

The data are presented as the mean standard deviation (SD) of three replicates of each group. Student’s *t*-test was used to compare values between the test and control groups. *p*values* < *0.05 indicated statistical significance. All calculations were performed using SPSS software version 13.0.

## Electronic supplementary material

Supplementary 1
