# Correction: LncRNA HCP5A promotes follicular thyroid carcinoma progression via miRNAs sponge

**DOI:** 10.1038/s41419-019-1868-7

**Published:** 2019-09-03

**Authors:** Leilei Liang, Jingchao Xu, Meng Wang, Gaoran Xu, Ning Zhang, Guangzhi Wang, Yongfu Zhao

**Affiliations:** Department of General Surgery, The Second Hospital of Dalian Medical University, Dalian, China

**Correction to: Cell Death & Disease**

10.1038/s41419-018-0382-7 Published online 07 March 2018

After publication of this article, it came to our attention that there was an error in Fig. 3a wherein the migration panel of pcDNA3.3 was replicated in the invasion panel for HCP5. The correct image for the invasion panel of HCP5 is provided in the corrected Fig. 3a below. This error did not impact the conclusions of the article. We apologize for any inconvenience this may have caused.

**Fig. 3:**
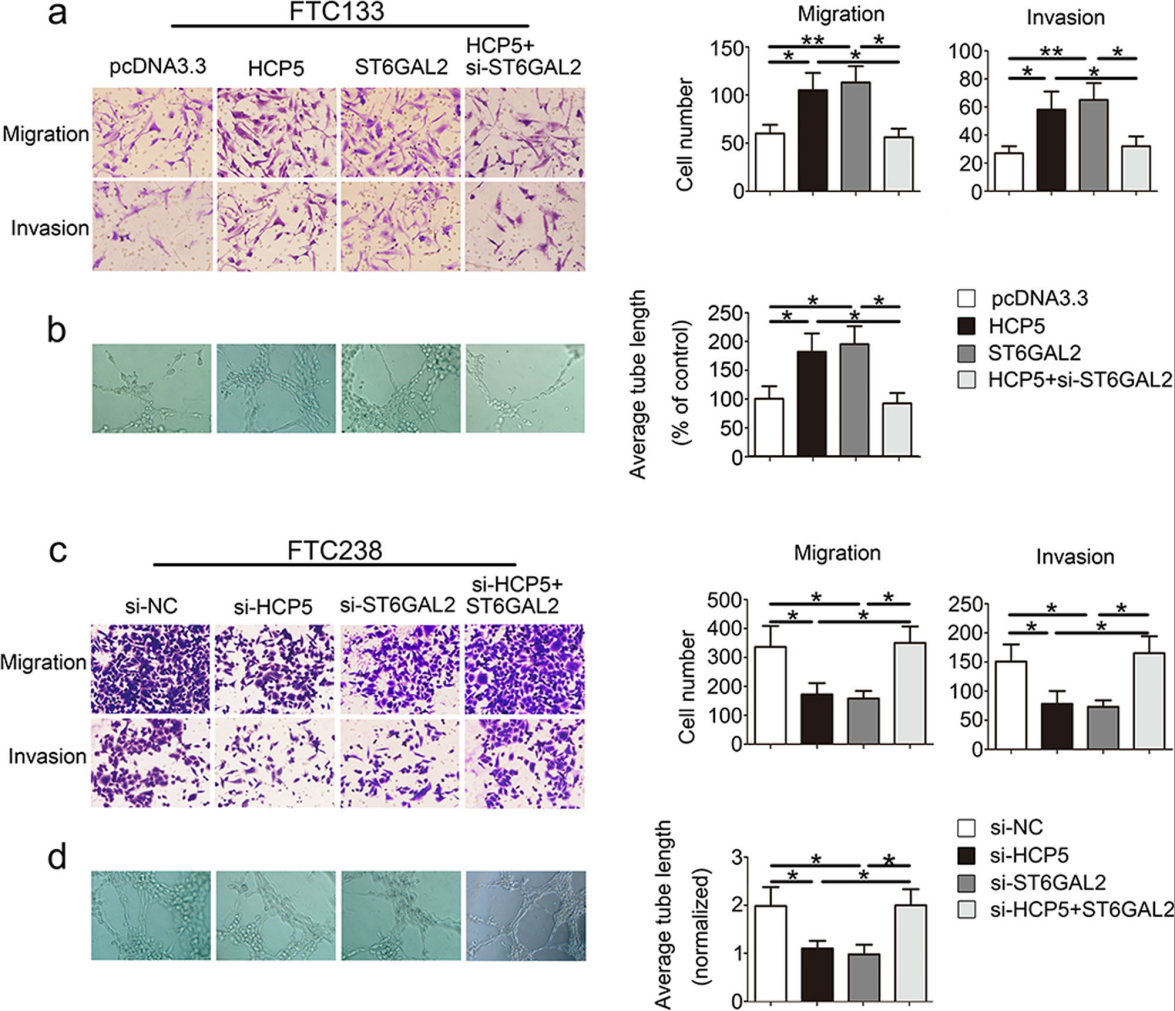
HCP5 regulates the migration, invasiveness and angiogenic ability of FTC cells. HCP5 can promote the development of FTC via enhancements in **a** migration, invasiveness and **b** angiogenic capacity of FTC cells, but **c**, **d** opposite results were found in the si-HCP5 and si-ST6GAL2 groups. (**p value  <  0.01, *p value <  0.05), scale bars: 20 μm)

This has been corrected in both the PDF and HTML versions of the Article.

