# Supplementary figures and images for: LncRNA HCP5 promotes follicular thyroid carcinoma progression via miRNAs sponge
Source: Cell Death Dis. 2018 Mar 7;9(3):372. doi: 10.1038/s41419-018-0382-7 (PMC5841368; doi:10.1038/s41419-018-0382-7)

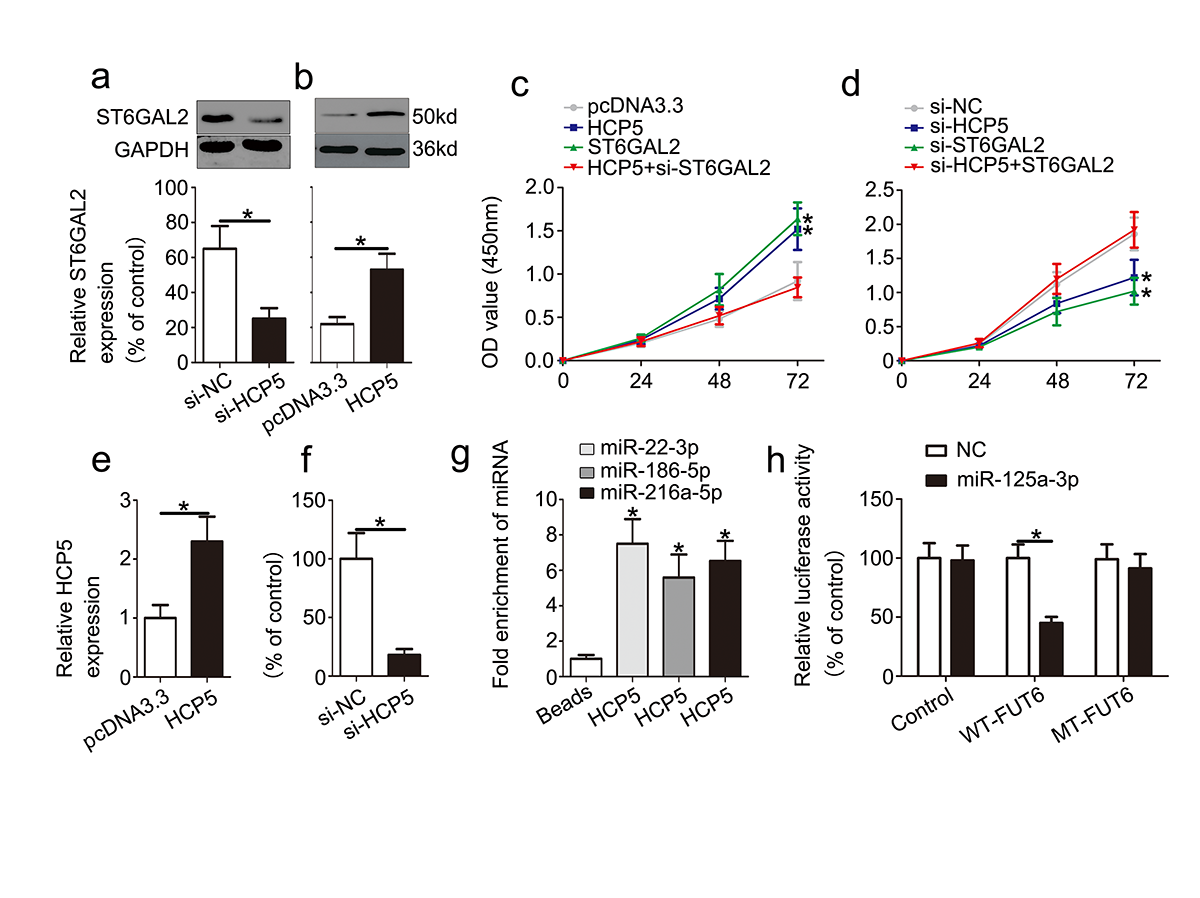

Supplement: Supplementary file 1 — Supplementary 1 [file 41419_2018_382_MOESM1_ESM.tif]
